# Pharmacological inactivation of PI3Kδ in the tumor microenvironment enhances efficacy of other immunotherapeutic agents

**DOI:** 10.1186/2051-1426-3-S2-P377

**Published:** 2015-11-04

**Authors:** Liang-Chuan S Wang, Holly Koblish, Michael Hansbury, Yue Zhang, Gengjie Yang, Timothy Burn, Paul Waeltz, Mark Rupar, Eddy Yue, Brent Douty, Thomas Maduskuie, Nikoo Falahatpisheh, Yun-Long Li, Andrew P Combs, Gregory Hollis, Reid Huber, Peggy Scherle

**Affiliations:** 1 Incyte Corporation, Wilmington, DE, USA

## 

Pharmacological inhibition of the oncogenic PI3Kδ pathway has been shown to be efficacious in patients with hematopoietic malignancies. However, its therapeutic application in patients with solid tumors has not yet been tested. Recently, genetic inactivation of PI3Kδ in mice was shown to delay the growth of solid tumors, resulting from the inactivation of Treg-mediated suppression of cytotoxic CD8^+^ T cell responses. Therefore we explored the immunotherapeutic potential of our PI3Kδ-selective compound, INCB050465, in multiple preclinical tumor models. We demonstrate that INCB050465 can block tumor growth in multiple established tumor models which are not dependent upon oncogenic PI3K signaling. Tumor growth inhibition is not observed in these models in immunocompromised mice, demonstrating that the anti-tumor effects of these agents require an intact immune system. To further investigate the immune-mediated mechanisms, tumors exposed to vehicle or INCB050465 were harvested and analyzed for modulation of gene expression and immune phenotype. INCB050465 was shown to significantly downregulate T cell gene signatures in tumors, and this was primarily due to depletion of CD4^+^CD25^+^FoxP3^+^ regulatory T cells. In contrast, the number of CD8^+^ T cells was shown to be higher in INCB050465-treated tumors. We next examined INCB050465 in combination with other immune modulators. The combination of PI3Kδ and JAK inhibition resulted in enhanced activity in a T cell-inflamed model by reducing both Treg and M2 macrophages, which then allowed the re-activation of both CD4 and CD8 T cells. In addition, PI3Kδ inhibition and PD-L1 blockade resulted in enhanced efficacy by depleting Treg and prolonging T cell responses over time. In summary, inactivation of PI3Kδ with a pharmacological inhibitor can enhance anti-tumor immunity by depleting Treg while increasing the numbers of cytotoxic CD8^+^ T cells. These data support clinical evaluation of the mechanism, and further studies to understand the molecular basis of efficacy and associated cellular responses may provide a rationale to identify individuals who may benefit most from PI3Kδ inhibitor-based immunotherapy combinations in the clinic.

